# The value of speckle-tracking echocardiography in identifying right heart dysfunction in patients with chronic thromboembolic pulmonary hypertension

**DOI:** 10.1007/s10554-018-1423-0

**Published:** 2018-07-30

**Authors:** Ai-Li Li, Zhen-Guo Zhai, Ya-Nan Zhai, Wan-Mu Xie, Jun Wan, Xin-Cao Tao

**Affiliations:** 1 Department of Cardiology, China-Japan Friendship Hospital, Beijing, 100029 China; 2 Department of Pulmonary and Critical Care Medicine, Center of Respiratory Medicine, China-Japan Friendship Hospital, Beijing, 100029 China; 3 National Clinical Research Center for Respiratory Diseases, Beijing, 100029 China

**Keywords:** Chronic thromboembolic pulmonary hypertension, Right ventricular function, Speckle tracking echocardiography, Strain

## Abstract

Right ventricular (RV) function is a significantly important factor in the determination of the prognosis of chronic thromboembolic pulmonary hypertension (CTEPH) patients. Speckle-tracking echocardiography (STE) is an angle-independent new technique for quantifying myocardial deformation that is capable of providing data on multiple parameters including longitudinal and transverse information of the myocardium. In the present study, we aimed to study the advantages of STE-derived parameters in identifying RV dysfunction in CTEPH patients. Sixty CTEPH patients (mean age: 55 years ± 13 years; 25 males) and 30 normal controls (mean age: 54 years ± 14 years; 14 males) were enrolled in this study. RV free wall (RVFW) systolic peak longitudinal strain (LS) including the basal, mid-, and apical-segments and the basal longitudinal and transverse displacement (basal-DL and basal-DT) were measured by STE. Global LS (GLS) of the RV was calculated by averaging the LS value of the 3 segments of RVFW. Clinical data of CTEPH patients were collected. CTEPH patients were divided into 2 subgroups according to the World Health Organization function classification. Clinical right heart failure (RHF) was defined as the presence of symptoms of heart failure and signs of systemic circulation congestion during hospitalization. The apical segment LS of the RVFW was lower than that in the basal and mid-segments in the control group (*P* < 0.001), but no significant difference was found among the 3 segments of LS in the CTEPH group (*P* = 0.263). When we used the cutoff value recommended by the American Society of Echocardiography guidelines to identify abnormal RV function, 30 CTEPH patients (50%) by tricuspid annular plane systolic excursion (TAPSE), 42 patients (70%) by fractional area change (FAC), 20 patients (33.33%) by RV index of myocardial performance (RVIMP), and 46 patients (77%) patients by GLS were determined to have abnormal RV function, respectively. Among multiple RV function indicators, TAPSE, FAC, GLS, basal-DL, and N-terminal pronatriuretic B-type natriuretic peptide showed significant differences between CTEPH patients with mild (WHO II) and severe symptoms (WHO III/IV) (all *P* < 0.001), while RVIMP and basal-DT showed no significant difference (*P* = 0.188 and *P* = 0.394, respectively). Pearson correlation analysis showed that GLS has no correlation with sPAP as evaluated by echocardiography in CTEPH patients (r = − 0.079, *P* = 0.574), and a weak to moderate correlation with RA area (r = 0.488, *P* = 0.000), the RV diameter (r = 0.429, *P* = 0.001), and the RVFW thickness (r = 0.344, *P* = 0.009). On receiver operating characteristic analysis, GLS has the largest area under the curve to identify RHF when the cutoff value was − 13.45%, the sensitivity was 78.2%, and the specificity was 84.6%, separately. Our study demonstrated that the depression of regional LS of RVFW is more pronounced in the basal and middle segments in CTEPH patients. Also, the longitudinal movement is much more important than the transverse movement when evaluating RV systolic function. As compared with conventional parameters, RVFW GLS showed more sensitivity to identify abnormal RV function and had the largest AUC for identifying RHF. Additionally, GLS showed no correlation with sPAP and a weak correlation with right heart morphological parameters in our CTEPH cohort.

## Introduction

Chronic thromboembolic pulmonary hypertension (CTEPH) is a disease characterized by pulmonary artery thromboembolism and vessel obstructive remodeling, with progressive increased pulmonary vascular resistance [1, 2]. In some patients, the extra pressure load may eventually lead to right ventricular (RV) dysfunction. Thus, accurate evaluation of RV function is important in the determination of the illness severity and prognosis of CTEPH patients. The hemodynamic parameters by right heart catheterization (RHC) and RV ejection fraction (EF) by cardiac magnetic resonance imaging (CMR) can provide prognostic information [1, 3]. However, these modalities are not always routinely used in continuous monitoring of RV function. Additionally, conventional echocardiographic parameters have been widely employed to roughly evaluate RV systolic function, but they have limitations [4]. Speckle-tracking echocardiography (STE) is an angle-independent new technique for quantifying myocardial deformation capable of providing data on multiple parameters including longitudinal and transverse information of the myocardium. Global longitudinal strain (GLS) of the RV free wall (RVFW) based on STE has been recommended by the American Society of Echocardiography (ASE) guidelines as a new parameter for estimating RV systolic function and has been reported to have prognostic value in heart failure [5] and pulmonary hypertension [6–8]. However, the characteristics and advantages of STE-derived parameters in assessing RV dysfunction in CTEPH patients have not been intensively studied thus far. We hypothesized that the use of multiple parameters based on STE could provide more comprehensive knowledge in the evaluation of RV dysfunction in CTEPH patients.

## Materials and methods

### Study population

We studied consecutive CTEPH patients at our hospital between November 2015 and December 2017 who had been diagnosed according to the diagnostic algorithms for CTEPH [1]. Patients with coronary artery disease, cardiomyopathies, significant left valvular disease (moderate to severe aortic or mitral stenosis or regurgitation), irregular heart rhythm, and/or poor image quality were excluded. In all, 65 CTEPH patients (mean age: 56 years ± 14 years; 26 males) were enrolled in the study. The clinical data of CTEPH patients were collected, including symptoms and signs, the level of N-terminal pronatriuretic B-type natriuretic peptide (NT-proBNP), and 6-min walk distance (6MWD) results. CTEPH patients were divided into 2 subgroups using the World Health Organization (WHO) function classification: patients with WHO II were designated as group A and those with WHO III/IV were designated as group B. Evidence of right heart failure (RHF) was defined as the presence of symptoms of heart failure and signs of systemic circulation congestion during hospitalization and was diagnosed by a cardiologist. CTEPH patients in our study were mostly treated with anticoagulants (100%) and diuretics (83%), and some patients were receiving endothelin receptor antagonists (12%), phosphodiesterase-5 inhibitors (30%), or their combination (4%), according to current guidelines. The control group was composed of 30 age- and gender-matched subjects (mean age: 54 years ± 14 years; 14 males) who had normal physical examination findings, no cardiopulmonary disease, and good image quality. All patients agreed to their data being used for research and the study protocol was approved by the hospital’s Human Research Ethics Committee (2016-92).

### Conventional echocardiography

Two-dimensional (2D) and Doppler echocardiography were performed using the Vivid E9 ultrasound system (General Electric Healthcare, Vingmed, Horten, Norway) by a senior doctor. Cardiac quantification was in accordance with the recommendations of the ASE guidelines [9]. Right heart morphological parameters included right atrial (RA) area, RV basal diameter, the ratio of RV and left ventricular (LV) basal diameter (RV/LV), proximal RV outflow diameter (RVOT prox), RVFW thickness, and pulmonary artery (PA) diameter. Conventional RV function parameters included tricuspid annular plane systolic excursion (TAPSE), RV fractional area change (FAC), and RV index of myocardial performance (RVIMP). Systolic pulmonary artery pressure (sPAP) was calculated by adding the tricuspid regurgitation (TR) peak gradient to the estimated RA pressure (RAP). RAP estimation was based on interrogation of the inferior vena cave (IVC) diameter and respiratory variation in the diameter of IVC and was scored as either 3, 8, or 15 mmHg [1].

### Two-dimensional speckle-tracking echocardiography

**Fig. 1:**
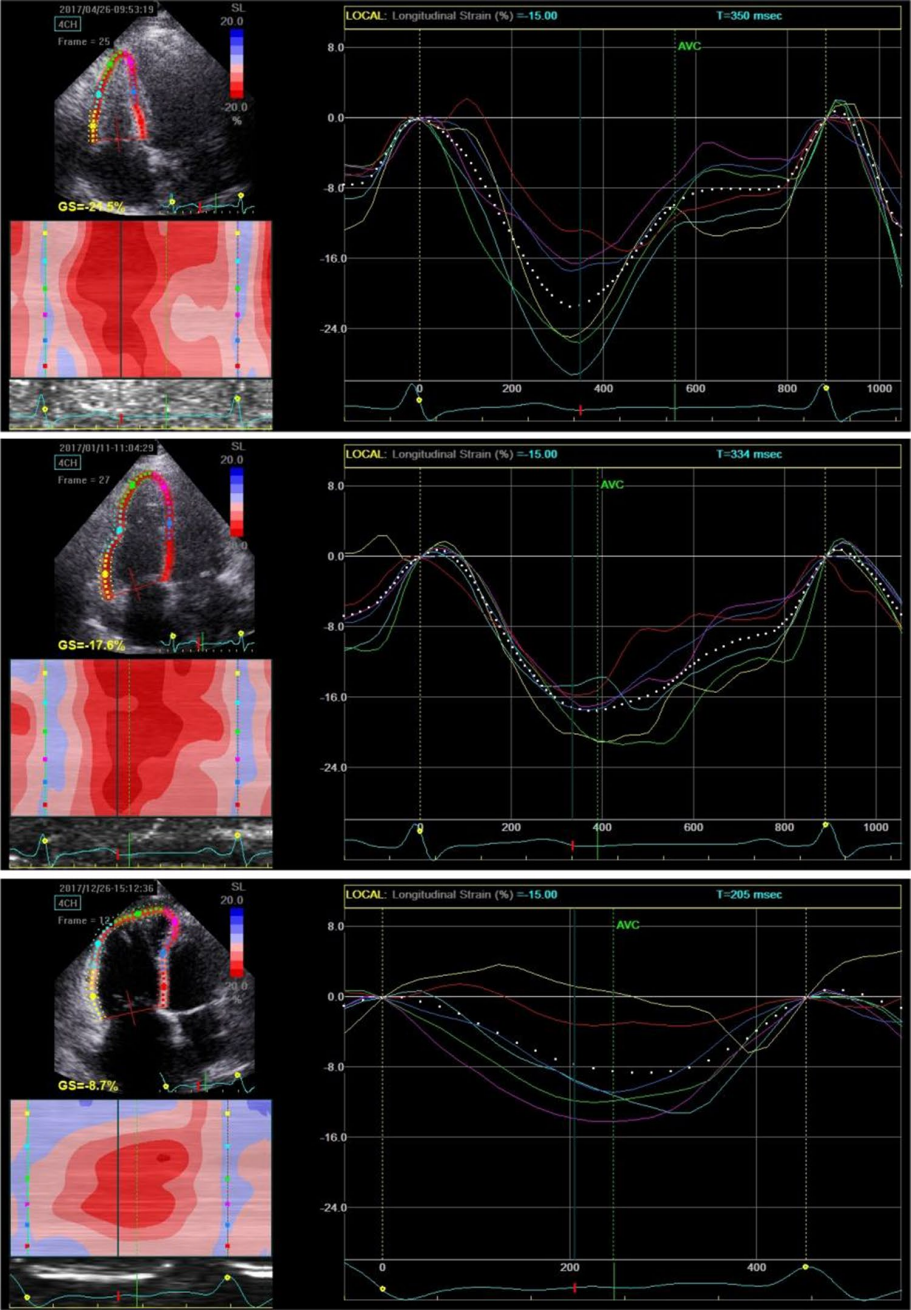
GLS and regional LS of the RV by STE. The 6 different colors represent the basal, mid-, and apical segments of the free wall and septum, respectively. Note the 3 segments of RVFW are on the left and the septum segments are on the right. GLS was calculated by averaging the value of the 3 segments of RVFW in our study. The upper image was from a normal subject (GLS = − 26.60%); the middle image was from a CTEPH patient with WHO II (GLS = − 19.45%); and the bottom image was from a CTEPH patient with WHO III (GLS = − 12.39%)

2D STE data for RV were obtained via the RV-focused apical 4-chamber view. Four cardiac cycles of RV imaging with a mean frame rate of 49 frames/s ± 8 frames/s were recorded for analysis. RVFW longitudinal strain (LS) was measured offline using the EchoPac software BT113 (General Electric Healthcare, Vingmed, Horten, Norway). The endocardial border of the RV was traced manually, and a region of interest (ROI) including the RVFW and septum was automatically generated, with the ROI width adjusted to a minimum. The RV myocardium was divided into 6 segments (basal-, mid-, and apical-segments of the free wall and the septum). The peak systolic LS was a negative percentage value, indicating tissue contraction/shortening (for convenience, we used the absolute value for analysis). We recorded the LS of the basal, mid-, and apical RVFW and calculated the RV GLS by averaging the value of the 3 segments (Fig. 1).

In the STE data, the longitudinal displacement (DL) and transverse displacement (DT) were also generated. We recorded the basal DL and DT of the RVFW for analysis. The mid- and apical-DT/DL values were excluded because of the small value and great dispersion.

The reproducibility of RV GLS, TAPSE, FAC, and RVIMP was assessed in a subgroup of 30 randomly chosen subjects. The correlation coefficients of interobserver variability for these were 0.683, 0.790, 0.533, and 0.583, respectively.

### Data analysis

Statistical analysis was performed with SPSS 19.0 (IBM Corp., Armonk, NY, USA). Continuous variables were presented as means ± standard deviations (SDs) or medians with interquartile ranges. Categorical variables were presented as numbers and percentages. Continuous variables between the 2 groups were compared by independent-sample *t* test for normal distribution data and the Mann–Whitney U test for abnormal distribution data. Categorical variables between the 2 groups were compared using Pearson’s Chi-Squared test. One-way analysis of variance and Student–Newman–Keuls q (SNK-q) test were used to compare the segmental strain of the RVFW. The Pearson correlation coefficients of sPAP, RAA, RVD, and RVFW thickness with GLS were tested in all subjects and only in the CTEPH group separately. Receiver operator characteristic (ROC) analysis was performed to compare the area under the curve (AUC) of RV function parameters to identify RHF in the CTEPH group. A 2-tailed *P* value of < 0.05 was considered to be statistically significant.

## Results

In all, 60 CTEPH patients (92.3%) and all normal subjects (100%) successfully underwent STE. Only 5 CTEPH patients were excluded from the analysis due to poor echogenicity. The baseline characteristics of the included CTEPH patients are summarized in Table 1.

**Table 1:** Baseline characteristics of the CTEPH study population

Characteristic	Overall (n = 60)
Age, year	55 ± 13
Sex (% male)	25 (42.67%)
WHO class II	28 (46.67%)
WHO class III, IV	32 (53.33%)
6MWD (m)	378.53 ± 63.07
NT-proBNP (pg/ml)	730 (282.93, 2132.50)
TR (moderate or greater)	25 (41.67%)
Pericardial effusion	13 (21.67%)
Right heart failure	19 (31.67%)
mPAP (mmHg)	46.39 ± 8.60
PVR (dyn s/cm^5^)	1101.20 ± 253.35
CI (l/min/m^2^)	1.89 ± 0.43

Data are presented as mean ± SD, median (interquartile range), or no. (%)

*WHO* World Health Organization, *6MWD* 6 min walk distance, *NT-proBNP* N-terminal pronatriuretic B-type natriuretic peptide, *TR* tricuspid valve regurgitation, *mPAP* pulmonary artery mean pressure, *PVR* pulmonary vascular resistance, *CI* cardiac index

**Fig. 2:**
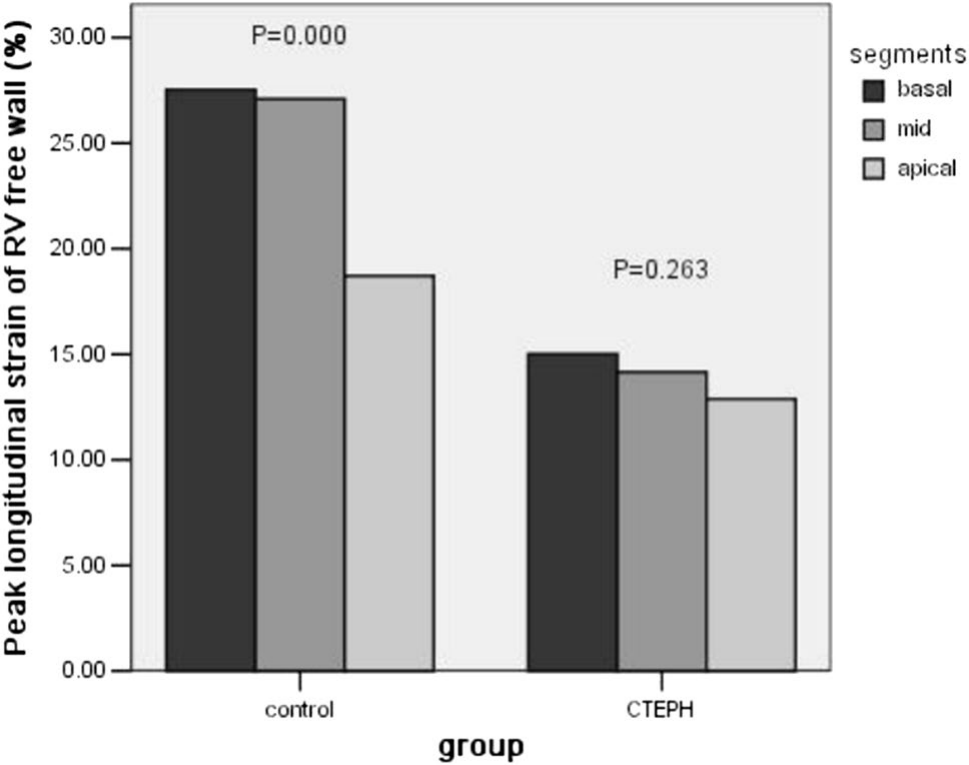
Segmental LS of the RVFW in the control and CTEPH groups. In the control group, LS in the apical segment was significant lower than that in the basal and mid-segments (*P* = 0.000). However, this LS gradient was lost in CTEPH patients; notably, no significant difference was found among the 3 segments of LS in the CTEPH group (*P* = 0.263)

As compared with the normal controls, CTEPH patients showed significant enlarged right heart dimension, increased RVFW thickness, dilated pulmonary arteries, and smaller LV size, with decreased RV function indicated by both conventional and STE-derived variables (Table 2). Unexpectedly, we found that the apical segment LS of the RVFW was lower than that in the basal and mid-segments in the control group patients, but this LS gradient was lost in CTEPH patients: no significant difference was found among the 3 segments of LS in the CTEPH group (Table 3; Fig. 2). This result means that the depression of RVFW segmental LS was more pronounced in the basal and middle segments in CTEPH patients. Furthermore, the value of basal DT was significantly smaller than that of basal DL both in the control group (9.73 mm ± 2.57 mm vs. 19.76 mm ± 4.46 mm, *P* = 0.000) and the CTEPH group (7.33 mm ± 2.38 mm vs. 14.24 mm ± 5.17 mm, *P* = 0.000). This result indicated that longitudinal movement is predominant in RVFW contraction.

**Table 2:** Comparison of conventional and STE echocardiographic parameters between the CTEPH and control groups

Variable	Control (n = 30)	CTEPH (n = 60)	*P* value
Age, years	54 ± 14	53 ± 13	0.562
Sex (% male)	14 (46.67%)	25 (42.67%)	0.159
RA area (cm^2^)	14.02 ± 2.46	25.28 ± 8.91	0.000
RV (mm)	35.21 ± 3.61	49.38 ± 6.25	0.000
RV/LV (mm)	0.78 ± 0.09	1.36 ± 0.30	0.000
RVFW thickness (mm)	3.56 ± 0.63	5.64 ± 1.45	0.000
RVOT (mm)	32.11 ± 2.01	37.18 ± 5.39	0.000
PA (mm)	26.19 ± 4.72	31.90 ± 5.85	0.000
sPAP (mmHg)	27.55 ± 4.06	84.18 ± 17.44	0.000
TAPSE (mm)	19.60 ± 1.73	16.21 ± 3.08	0.000
FAC (%)	44.17 ± 8.28	29.14 ± 7.95	0.000
RVIMP	0.36 ± 0.08	0.47 ± 0.18	0.000
LVDd	48.55 ± 3.97	40.88 ± 4.68	0.000
LVEF (%)	64.72 ± 3.63	67.58 ± 5.71	0.183
STE-derived parameters			
Basal-LS (%)	26.10 ± 5.34	15.68 ± 6.63	0.000
Mid-LS (%)	26.45 ± 4.77	14.78 ± 6.42	0.000
Apical-LS (%)	18.99 ± 5.82	13.22 ± 4.93	0.000
GLS (%)	24.04 ± 4.38	14.56 ± 5.54	0.000
Basal-DL (mm)	19.29 ± 4.30	14.87 ± 5.06	0.001
Basal-DT (mm)	9.75 ± 2.72	7.43 ± 2.56	0.001

Data are presented as mean ± SD, or no. (%)

*RA* right atria, *RV* right ventricular, *RVOT* right ventricular outflow diameter, *PA* pulmonary artery, *sPAP* systolic pulmonary artery pressure, *TAPSE* tricuspid annular plane systolic excursion, *FAC* fractional area change, *RVIMP* right ventricular index of myocardial performance, *LVDd* left ventricular diameter (diastole), *LVEF* left ventricular ejection fraction, *LS* longitudinal strain, *GLS* global longitudinal strain, *DL* longitudinal displacement, *DT* transverse displacement

**Table 3:** Comparison of different segmental strains of the RVFW

Group	Basal	Mid	Apical	*F* value	*P* value
RVFW LS (%)					
Control group	26.11 ± 5.34	26.45 ± 4.77	18.99 ± 5.83	17.647	0.000
CTEPH group	15.68 ± 6.63	14.78 ± 6.42	13.22 ± 4.93	1.707	0.186

Data are presented as mean ± SD

*LS* longitudinal strain

Interestingly, when we used the cutoff value recommended by the ASE guidelines to identify abnormal RV function, we found 30 CTEPH patients (50%) by TAPSE (< 17 mm), 42 patients (70%) by FAC (< 35%), 20 patients (33.33%) by RVIMP (> 0.54), and 46 patients (77%) by GLS (< 20%) to have abnormal RV function. This means that RV GLS identifies a higher number of patients with impaired RV systolic function as compared with conventional measurements.

When comparing echocardiographic and clinical variables in mild and severe symptom CTEPH patients, we found that WHO III/IV patients had more enlarged right hearts, but that there was no significant difference in RVFW thickness, PA diameter, or pulmonary artery pressure between the subgroups. Among multiple RV function indicators, TAPSE, FAC, GLS, basal-DL, and NT-proBNP showed significant differences, while RVIMP and basal-DT did not (Table 4). These results remind us that RVIMP may be not a good parameter to identify severe RV dysfunction, and the depression of longitudinal function may be more predominant in CTEPH patients.

**Table 4:** Comparison of echocardiographic and clinical variables between the subgroups of CTEPH patients

Variable	CTEPH-A group (n = 28)	CTEPH-B group (n = 32)	*P* value
Age, year	51.96 ± 10.23	58.47 ± 15.90	0.137
RA area (cm^2^)	21.84 ± 5.67	30.22 ± 10.57	0.003
RV (mm)	47.71 ± 6.28	51.86 ± 6.07	0.028
RV/LV (mm)	1.25 ± 0.19	1.51 ± 0.32	0.005
RVFW thickness (mm)	5.20 ± 1.10	6.08 ± 1.63	0.105
RVOT (mm)	35.54 ± 5.18	40.65 ± 4.93	0.002
PA (mm)	31.75 ± 6.80	32.50 ± 4.31	0.661
sPAP (mmHg)	79.39 ± 18.67	84.88 ± 16.38	0.598
TAPSE (mm)	18.11 ± 1.48	14.58 ± 3.11	0.000
FAC (%)	34.32 ± 5.14	24.17 ± 7.06	0.000
RVIMP	0.44 ± 0.19	0.52 ± 0.16	0.188
LVDd (mm)	41.90 ± 4.61	39.74 ± 4.59	0.145
LVEF (%)	68.24 ± 5.12	66.84 ± 5.54	0.342
Basal-LS (%)	17.89 ± -5.03	12.00 ± 4.73	0.001
Mid-LS (%)	16.87 ± 6.07	11.17 ± 5.60	0.002
Apical-LS (%)	14.82 ± 5.03	10.69 ± 3.50	0.002
GLS (%)	16.53 ± 5.08	11.33 ± 4.55	0.000
DLbase (mm)	17.11 ± 4.95	11.59 ± 3.51	0.001
DTbase (mm)	7.64 ± 2.43	7.04 ± 2.48	0.394
TR (moderate or greater)	7 (25%)	18 (56.25%)	0.014
Pericardial effusion	2 (7.14%)	11 (40.74%)	0.011
6MWD (m)	391.5 ± 68.06	363.71 ± 58.28	0.415
NT-proBNP (pg/ml)	385 (141, 730)	1925 (782, 3753)	0.001
mPAP by RHF (mmHg)	47.43 ± 5.94	48.17 ± 4.88	0.695
PVR	991.60 ± 301.08	1170.95 ± 202.41	0.148
CI	2.24 ± 0.45	1.67 ± 0.22	0.002

Data are presented as mean ± SD, or no. (%)

*RA* right atria, *RV* right ventricular, *RVOT* right ventricular outflow diameter, *PA* pulmonary artery, *sPAP* systolic pulmonary artery pressure, *TAPSE* tricuspid annular plane systolic excursion, *FAC* fractional area change, *RVIMP* right ventricular index of myocardial performance, *LVDd* left ventricular diameter (diastole), *LVEF* left ventricular ejection fraction, *LS* longitudinal strain, *GLS* global longitudinal strain, *DL* longitudinal displacement, *DT* transverse displacement, *TR* tricuspid valve regurgitation, *6MWD* 6 min walk distance, *NT*-proBNP N-terminal pronatriuretic B-type natriuretic peptide, *mPAP* pulmonary artery mean pressure, *RHC* right heart catheterization, *PVR* pulmonary vascular resistance, *CI* cardiac index

**Fig. 3:**
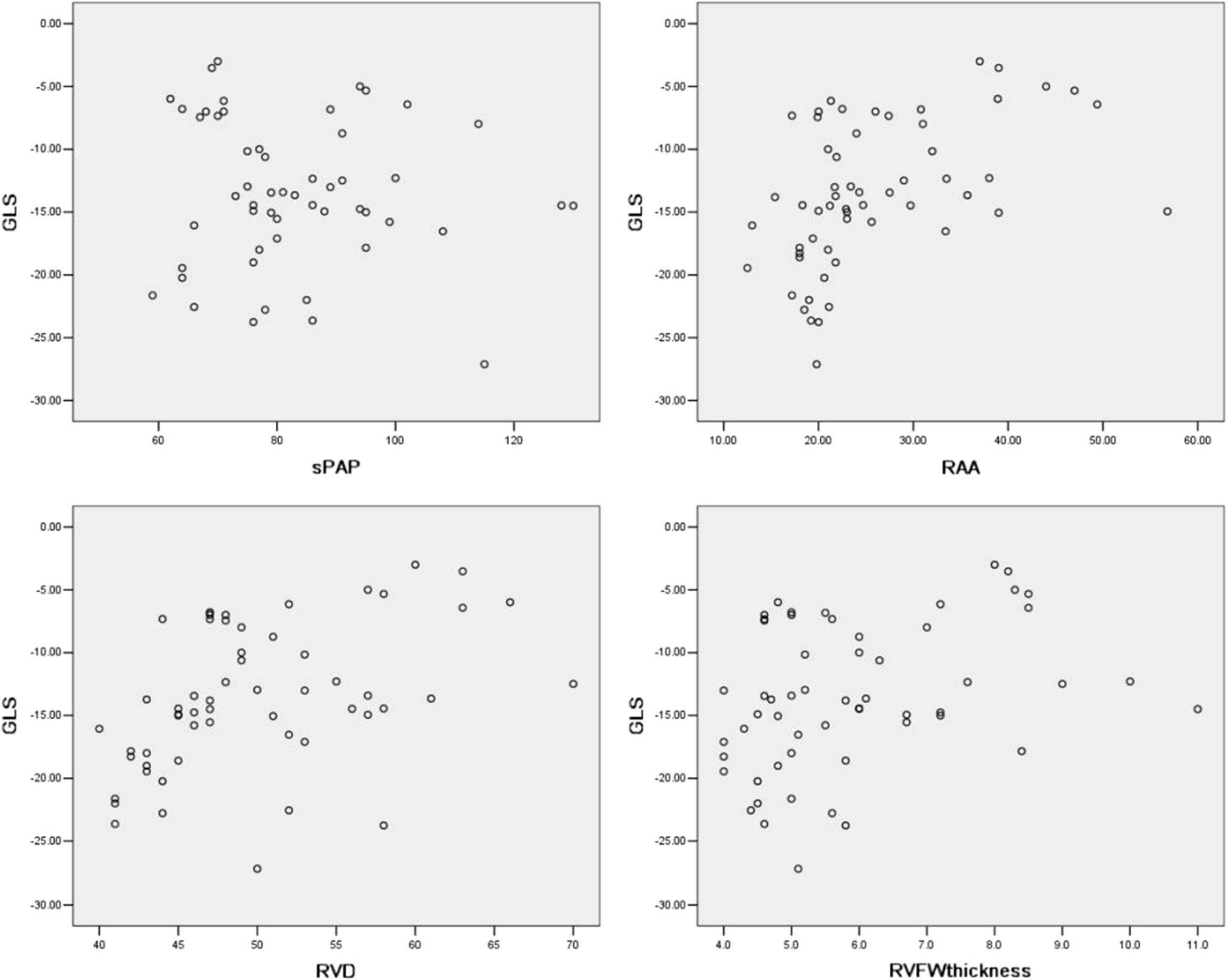
Pearson correlation analysis showed GLS had no correlation with sPAP as evaluated by echocardiography in CTEPH patients (r = − 0.079, *P* = 0.574) and a weak to moderate correlation with RAA (r = 0.488, *P* = 0.000); RVD (r = 0.429, *P* < 0.001); and RVFW thickness (r = 0.344, *P* = 0.009)

According to Pearson correlation analysis (Fig. 3), we found that GLS (not the absolute value) of RVFW has a correlation with sPAP evaluated by echocardiography (r = − 0.493, *P* < 0.0001); RAA (r = 0.615, *P* < 0.0001); RVD (r = 0.702, *P* < 0.0001); and RVFW thickness (r = 0.493, *P* < 0.0001) when including all subjects (both control and CTEPH groups). However, if only CTEPH patients were included, then GLS showed no correlation with sPAP (r = − 0.079, *P* = 0.574) and a weak to moderate correlation with RAA (r = 0.488, *P* = 0.000); RVD (r = 0.429, *P* < 0.001); and RVFW thickness (r = 0.344, *P* = 0.009).

**Fig. 4:**
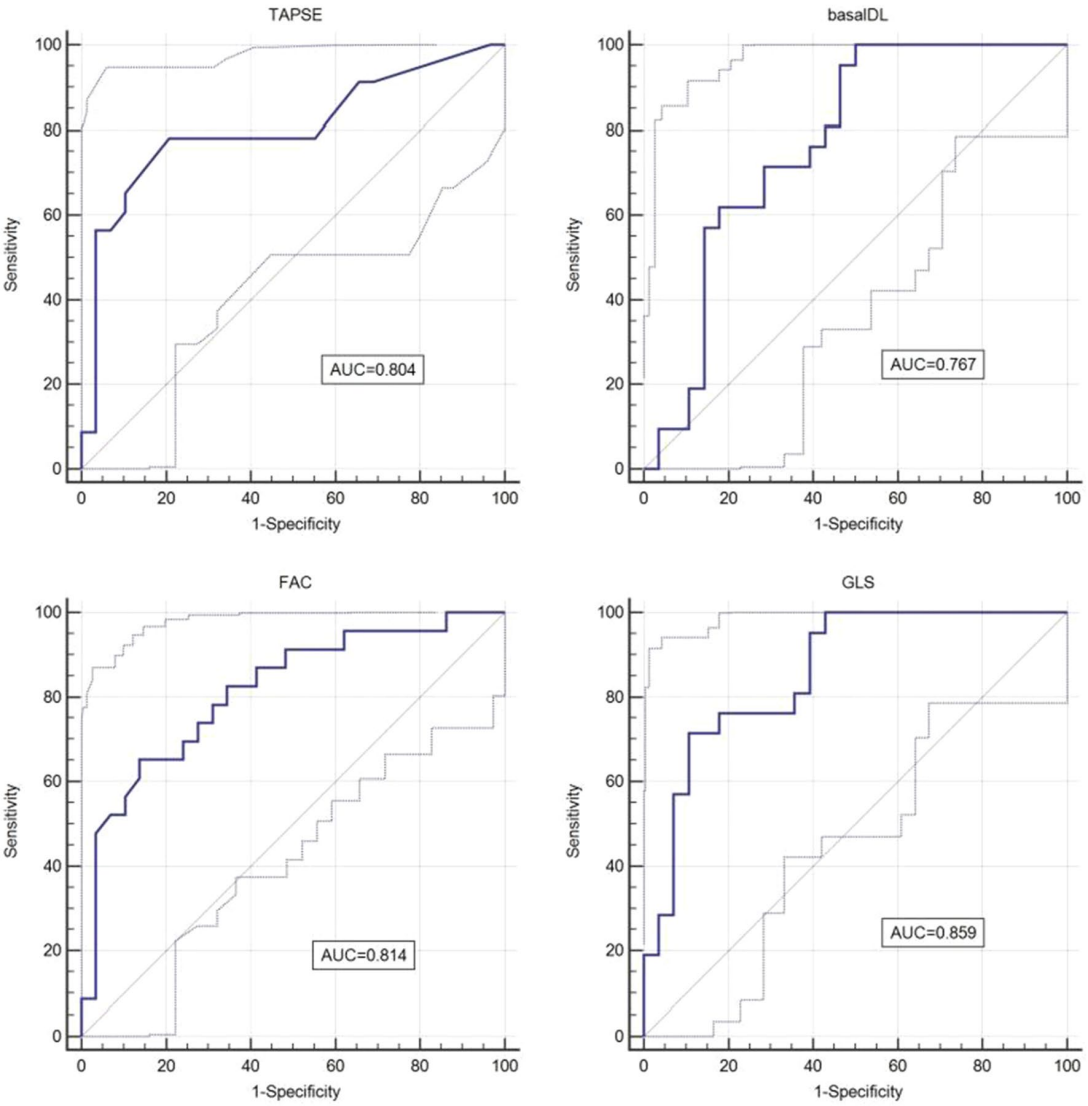
ROC analysis revealed the AUC GLS (0.859), FAC (0.814), TAPSE (0.804), and basal-DL (0.767) for identifying RHF

The results of ROC analysis are showed in Table 5 and Fig. 4. GLS has the largest AUC to identify RHF, when the cutoff value was − 13.45%, the sensitivity was 78.2%, and the specificity was 84.6%, respectively. Basal-DT showed the smallest AUC and thus cannot be used to identify RHF (*P* = 0.263).

**Table 5:** Results of ROC analysis

	AUC	*P* value	Cutoff value	Sensitivity (%)	Specificity (%)
TAPSE (mm)	0.804	< 0.0001	≤ 16	78.3	79.3
FAC (%)	0.814	< 0.0001	≤ 25.6	65.2	86.2
RVIMP	0.715	< 0.01	> 0.58	47.1	91.3
GLS (%)	0.859	< 0.0001	> − 13.45	78.2	84.6
Basal-DL (mm)	0.767	0.0001	> − 14.55	76.19	60.71

## Discussion

STE imaging is a new method that provides speckle tracking of natural acoustic markers in myocardium; as such, it is angle-independent and not influenced by heart translational motion, which is a potential concern with TAPSE by M-mode measures. STE-derived parameters include both LS/displacement and DT. GLS of RVFW has shown a good correlation with RVEF by CMR [8] and has been recommended by the ASE guidelines as a new parameter for estimating RV systolic function [9]. However, STE-derived parameters have not been fully substantiated in some clinical settings. Our study reports the value of STE-derived parameters for estimating RV dysfunction in CTEPH patients.

The main findings of our study are as follows. First, STE-derived parameters can evaluate the RVFW systolic contraction in a more comprehensive way. According to the analysis of the regional and global STE-derived parameters, we found that the depression of regional LS of RVFW is more pronounced in the basal and middle segments in CTEPH patients. Also, the longitudinal movement is much more important than the transverse movement when evaluating RV systolic function.

Second, although both conventional and STE-derived parameters can detect RV dysfunction in CTEPH patients, the sensitivity, repeatability, and value of prediction of RHF are different. GLS showed more sensitivity to identifying abnormal RV function with a cutoff value of − 20% as compared with conventional parameters and had the largest AUC for identifying RHF. The inter-observer variability of GLS is acceptable (better than that of FAC).

Third, GLS showed no correlation with sPAP and a weak correlation with right heart morphological parameters in our CTEPH cohort. These findings remind us that, in CTEPH patients with RV dysfunction, the level of pulmonary artery pressure can not completely and truly reflect the degree of impaired RV function.

### Right-heart maladaptive remodeling and characteristics of right ventricular free wall motion in chronic thromboembolic pulmonary hypertension patients

CTEPH patients had significant morphological and functional changes in our study, characterized by right heart enlargement and RV dysfunction. Because of pulmonary artery thromboembolism and vessel obstructive remodeling, pulmonary vascular resistance and RV afterload increased progressively in CTEPH patients. The ventricles adapt to the increased afterload by increasing wall thickness and myocardial contractility. Due to the smaller thickness and high compliance of the RV wall, the compensatory ability is often insufficient and maladaptive remodeling is more likely to occur in the right heart [10]. Our study was in agreement with this view.

In our study, we found that the depression of RVFW strain in CTEPH patients was more pronounced in the basal and middle segments. Sunbul et al. reported similar results when comparing RV longitudinal deformation parameters and exercise capacity in CTEPH patients [11]. These findings suggested that the response to increased afterload may not be the same in different segments of the RVFW. The myocardial impairment of the basal-mid RVFW may contribute more to RV dysfunction. In the typical echocardiographic signs of acute pulmonary embolism (e.g., afterload increased suddenly), the hypokinesis of the basal-mid RVFW with normal contraction of the apical segment seems to confirm this point of view [12]. Moreover, RV myocardium consists of 2 layers of circumferential subepicardium myofibers and longitudinal subendocardial myofibers [13]. Previous studies has been reported that longitudinal but not circumferential deformation reflects global RV contractile function by using multilayer strain analysis [14] and open pericardium animal modeling [15]. This is confirmed by our study. Based on these contractile characteristics of RV myocardium, we think that it is reasonable to use the basal-mid segmental longitudinal function parameters to evaluate RV systolic function, especially when the apical segment cannot be clearly displayed in enlarged RV.

### Advantages of speckle-tracking echocardiography-derived global longitudinal strain in identifying RV dysfunction in chronic thromboembolic pulmonary hypertension patients

We found that TAPSE, FAC, RVIMP, and STE-derived parameters all are capable of indicating RV dysfunction in CTEPH patients. This was consistent with findings reported in other studies that compared echocardiographic parameters to CMR parameters [16–18]. However, the sensitivity and practical value of each of these parameters may be different. GLS can identify more patients with abnormal RV function with a cutoff value of − 20%, indicating more sensitivity as compared with conventional parameters. ROC analysis showed that GLS has the largest AUC (0.859) for identifying RHF with a cutoff value of − 13.45%, while FAC takes second place (AUC = 0.804). Measurement of FAC depends heavily on endocardium identification and its repeatability was the lowest in our study. The success of STE analysis also depends on good imaging quality, but the tracing process is automatic, so the interobserver variability is better than that of FAC. Additionally, STE-derived basal-DL had a similar AUC to that of TAPSE for identifying RHF, but it has the advantages of angle-independency and not being influenced by heart translational motion.

TAPSE is easy to measure and not heavily dependent on image quality, so the repeatability was obviously better than other indexes. But it only represents the annular longitudinal movement of RVFW and slightly less sensitive to identify impaired RV function than FAC and GLS. In our study, RVIMP has the lowest sensitivity and smallest AUC to identify abnormal RV function, especially in severe patients with RHF. This may be due to the elevated RA pressure that shorten the isovulumetric relaxation period.

Based on these findings, we believed that STE-derived longitudinal global and regional parameters had advantages in identifying RV dysfunction in clinical practice.

### Correlation of global longitudinal strain with systolic pulmonary artery pressure by echocardiography and right ventricular remodeling parameters

We found GLS does have correlations with sPAP by echocardiography and right heart remodeling parameters if we included all control and CTEPH patients, and RV basal diameter showed the largest correlation coefficient. However, unexpectedly, when we only included CTEPH patients for analysis, there was no correlation between GLS and sPAP. This is somewhat different from the reports of other studies. Wright et al. [19] studied 187 pulmonary arterial hypertension (PAH) patients at two time points. They found that there was a highly significant correlation between Δ RVFW Strain and Δ PASP. But in a subset of patients (n = 64), RVFW strain showed no significant correlation with invasive PASP at visit 2 time points(*r*=-0.23, *P* = 0.08). Li et al. [20] studied 66 pulmonary hypertension patients (41 were CTEPH patients, 65% patients with WHO I/II), they found that RVFW LS had a positive correlation with mPAP by RHC (*r* = 0.597, *P* < 0.001). The conditions of the CTEPH patients included in our study were severe, including 53% patients with heart function of WHO III/IV, and this may explain this result. As we know, pulmonary artery pressure is determined by the cardiac output and the pulmonary vascular resistance. When RV function decreases obviously, the output of the right heart drops, and the measured pulmonary arterial pressure will not increase even if the pulmonary vascular resistance increases with the progression of the disease. It reminds us that, in clinical practice, it is the morphological and functional parameters but not the level of pulmonary artery pressure that reflects the degree of impaired RV function.

### Study limitations

There are few limitations of our study. First, it is a single-center study and its sample size is relatively small because CTEPH is not a common disease. Second, we did not include pulmonary thromboembolism patients whose pulmonary artery pressure was slightly elevated (35–50 mmHg by echocardiography). Whether STE-derived GLS can detect subclinical abnormal RV function or not is unknown in this cohort; however, this question is interesting and we hope to study it in the near future. Third, not all patients underwent RHC during the time of this study; some of the data of RHC were collected from previous records. Considering the long time interval between RHC and echocardiography, we used the sPAP evaluated by Doppler echocardiography but not mPAP obtained by RHC for correlation analysis, and this is not the ideal choice. Finally, RV strain analysis was unable to evaluate the outlet portion function, though this area is considered as a lesser contributor to the overall RV function. Maybe 3-dimensional RVEF can compensate for this.

## Conclusions

Our study demonstrated that STE-derived parameters can evaluate the RVFW systolic contraction in a more comprehensive way. The depression of regional LS of RVFW is more pronounced in the basal and middle segments in CTEPH patients. Also, the longitudinal movement is much more important than the transverse movement when evaluating RV systolic function. As compared with conventional parameters, RVFW GLS showed more sensitivity to identifying abnormal RV function with a cutoff value of − 20% and had the largest AUC for identifying RHF. Additionally, GLS showed no correlation with sPAP and a weak correlation with right heart morphological parameters in our CTEPH cohort.
